# Procyanidin B2 inhibits the activation of hepatic stellate cells and angiogenesis via the Hedgehog pathway during liver fibrosis

**DOI:** 10.1111/jcmm.14543

**Published:** 2019-07-21

**Authors:** Jiao Feng, Chengfen Wang, Tong Liu, Jingjing Li, Liwei Wu, Qiang Yu, Sainan Li, Yuting Zhou, Jie Zhang, Jiaojiao Chen, Jie Ji, Kan Chen, Yuqing Mao, Fan Wang, Weiqi Dai, Xiaoming Fan, Jianye Wu, Chuanyong Guo

**Affiliations:** ^1^ Department of Gastroenterology, Putuo People's Hospital Tongji University School of Medicine Shanghai China; ^2^ Department of Gastroenterology, Shanghai Tenth People's Hospital Tongji University School of Medicine Shanghai China; ^3^ Shanghai Tenth Hospital School of Clinical Medicine of Nanjing Medical University Shanghai China; ^4^ Department of Gerontology, Shanghai General Hospital Shanghai Jiaotong University School of Medicine Shanghai China; ^5^ Department of Oncology, Shanghai General Hospital Shanghai Jiaotong University School of Medicine Shanghai China; ^6^ Department of Gastroenterology Zhongshan Hospital of Fudan University Shanghai China; ^7^ Shanghai Institute of Liver Diseases Zhongshan Hospital of Fudan University Shanghai China; ^8^ Department of Gastroenterology Jinshan Hospital of Fudan University Jinshan, Shanghai China

**Keywords:** angiogenesis, Hedgehog pathway, hepatic stellate cells, liver fibrosis, procyanidin B2

## Abstract

**Background:**

Liver fibrosis is a wound‐healing process of liver featured by the over‐deposition of extracellular matrix (ECM) and angiogenesis. However, the effective treatment is lacking. Procyanidin B2 (PB2) is a flavonoid extract abundant in grape seeds with anti‐oxidant, anti‐inflammatory and anti‐cancer properties. The present study aimed to determine effects of PB2 on liver fibrosis.

**Method:**

The CCl4‐induced mouse liver fibrosis model and a human hepatic stellate cell (HSC) line (LX2 cells) were used to study the activation, ECM production and angiogenesis of HSCs through Western blotting analysis, immunohistochemistry, immunofluorescence staining, flow cytometry and tubulogenesis assay. A Hedgehog (Hh) pathway inhibitor (cyclopamine) and Smoothened agonist (SAG) were used to investigate the role of PB2 on Hh pathway.

**Results:**

The results showed that PB2 could inhibit the proliferation and induce apoptosis of HSCs. PB2 could also down‐regulate the expressions of VEGF‐A, HIF‐1α, α‐SMA, Col‐1 and TGF‐β1 of HSCs in vivo and in vitro. The application of SAG and cyclopamine proved that PB2 targets on Hh pathway.

**Conclusions:**

PB2 inhibited the Hh pathway to suppress the activation, ECM production and angiogenesis of HSCs, therefore reverses the progression of liver fibrosis in vivo and in vitro.

## 1 INTRODUCTION

Liver fibrosis is a wound‐healing process of the liver in response to repeated and chronic liver injuries, including viral infection, cholestatic liver disease, alcohol abuse, non‐alcoholic steatohepatitis, non‐alcoholic fatty liver disease and the side effects of some drugs.^1^, ^2^, ^3^ It is characterized by the excessive deposition of extracellular matrix (ECM) and formation of fibrous scar, giving rise to hepatic dysfunction and the possible progression to liver cirrhosis and hepatocellular carcinoma.^4^ Fortunately, liver fibrosis is a reversible process if the primary causative factors are removed, but this is always difficult and time‐consuming. Currently, the most effective therapeutic treatment for liver fibrosis is liver transplantation.^3^ Liver transplantation is not available and popular, however, because of the limitation of organ donors. Therefore, there is need to identify a new agent for the treatment of liver fibrosis to relieve liver dysfunction and decrease complications.

The pathogenesis of liver fibrosis has recently been well‐described, and hepatic stellate cells (HSCs) activation is the critical stage.^4^, ^5^ In healthy livers, HSCs are liver mesenchymal cells that are responsible for vitamin A storage. HSCs are localized in Disse's space and have a quiescent phenotype (qHSCs); however, when the liver is challenged with an injury stimulus, the Kupffer cells and other mesenchymal cells are activated and secrete various fibrogenic cytokines, including transforming growth factor‐beta 1 (TGF‐β1), platelet‐derived growth factor (PDGF) and epidermal growth factor (EGF), which result in the activation of HSCs.^5^ The activated phenotype of HSCs (aHSCs) then undergoes a cascade of activation responses, leading to the production of excessive ECM and fibrogenesis.

Except for the deposition of ECM, liver injury is associated with angiogenesis. Further, angiogenesis and fibrogenesis always develop in parallel with chronic liver disease because of tissues hypoxia.^6^, ^7^ Pathological angiogenesis exacerbates structure turbulence instead of providing relief. It has been reported that inhibition of angiogenesis by tyrosine kinase inhibitors, such as sunitinib or sorafenib, improves liver fibrosis in rodents.^8^, ^9^ Hence, angiogenesis is also an important target for the treatment of liver fibrosis.

Procyanidins are a type of flavonoid that are widespread in red wine, green and black tea, cocoa/chocolate, and fruit juices.^10^, ^11^ Procyanidins have been shown to possess anti‐oxidant, anti‐tumour, anti‐inflammatory, anti‐allergic, and cardiovascular and brain protection properties.^12^, ^13^, ^14^ Among the procyanidins, a B‐type procyanidin dimer, procyanidin B2(PB2), has been shown to possess greater anti‐oxidant and anti‐tumour effects than other PAs, such as procyanidin B1, B4 and B5.^15^, ^16^ Therefore, the special bioactivities of PB2 aroused our interest. Justino et al have reported that the *Annona crassiflora* fruit peel extract, which contains PB2, exhibits hepatoprotective properties.^17^ Yang et al showed that PB2 can protect against carbon tetrachloride (CCl4)‐induced acute liver injury in mice via suppression of the inflammatory response and apoptosis.^18^ There are few studies involving the effects of PB2 on chronic liver injuries, including the underlying mechanism of action.

The Hedgehog (Hh) pathway is responsible for embryogenesis, development and tissue remodelling.^19^ The Hh pathway plays an important role during various liver injuries, such as liver fibrosis, inflammation‐related injury and liver carcinogenesis.^6^, ^20^ Activation of HSCs and angiogenesis is associated with the Hh signalling pathway.^1^, ^21^ Targeting the Hh pathway has become a promising treatment for liver fibrosis.^1^ Therefore, the study was designed to examine the effects of PB2 on liver fibrosis, with particular attention to the activation of HSCs, angiogenesis and the Hh pathway.

## 2 MATERIALS AND METHODS

### 2.1 Reagents

PB2, pentobarbital sodium salt, cyclopamine hydrate and Smoothened agonist (SAG) were purchased from Sigma‐Aldrich (Merck KGaA). Sorafenib tosylate was purchased from Selleck Chemicals. CCl4 was purchased from China Sinopharm International Corporation. TGF‐β1 was obtained from PeproTech. Alanine aminotransferase (ALT), aspartate aminotransferase (AST) and hydroxyproline detection kits were purchased from Nanjing Jiancheng Bioengineering Institute. Oligonucleotide primers were synthesized by Generay Biotech Co., Ltd. The PrimeScript RT Reagent kit and SYBR Premix Ex Taq were purchased from TaKaRa Biotechnology. The primary antibodies used in the study are listed in Table 1. Anti‐goat or antimouse secondary antibodies were obtained from Dako. Dulbecco's Modified Eagle Medium (DMEM) and foetal bovine serum (FBS) were from HyClone (GE Healthcare). The Annexin V‐FITC apoptosis detection kit was purchased from BD Biosciences. Growth factor‐reduced Matrigel was purchased from BD Biosciences. PB2, sorafenib and cyclopamine were dissolved in DMSO (<0.1% [v/v]) for in vitro treatment.

**Table 1:** The primary antibodies used in the study

Antibody	Species	Targeted species	Dilution ratio	Supplier	Catalogue number
HIF‐1α	R	H	1:1000	PT	20960‐1‐AP
HIF‐1α	M	H, M	1:1000	Abcam	ab113642
α‐SMA	R	H, M	1:1000	PT	14395‐1‐AP
Col‐1	R	H, M	1:1000	PT	14695‐1‐AP
CD31	R	H, M	1:500	Abcam	ab28364
VEGF‐A	M	H, M	1:1000	Abcam	ab1316
SMO	R	H, M	0.1‐1 µg/mL	Abcam	ab72130
GLI1	M	H	1:1000	CST	2643
GLI1	R	H, M	1:1000	Abcam	ab151796
β‐actin	M	H, M, R	1:1000	CST	3700
TGF‐β1	R	H, M, R	1:500	BT	BS1361

Abbreviations: H, human; M, mouse; R, rabbit; CST, Cell Signaling Technology (Danvers, MA, USA). PT (Chicago, IL, USA). Abcam (Cambridge, MA, USA). BT, Bioworld Technology (St. Louis Park, MN, USA).

### 2.2 Animals and establishment of CCl4‐induced liver fibrosis model

Six‐week‐old male C57 mice weighing 24 ± 2 g were purchased from Shanghai SLAC Laboratory Animal Co., Ltd. and kept in environmentally controlled conditions with free access to standard laboratory chow and water. The experiments were approved by the Animal Care and Use Committee of Shanghai Tongji University and conducted following the ARRIVE Guidelines. Forty‐two mice were randomly divided into seven groups (n = 6), as follows: (a) normal control (NC) group, no treatment; (b) vehicle group, injected with peanut oil intraperitoneally only; (c) PB2 group, treated with 150 mg/kg of PB2 by gavage during the 5th‐8th weeks; (d) CCl4 group, injected with CCl4; and (e‐g) L, M and H groups, respectively, treated with CCl4 and PB2 (50, 100 and 150 mg/kg, respectively).^18^ The CCl4‐induced liver fibrosis model was established according to previous studies.^22^, ^23^, ^24^, ^25^, ^26^

To furtherly investigate the effect of PB2 on Hh pathway in vivo, another 20 mice were induced by CCl4 to establish the liver fibrosis model and then randomly divided into four groups (n = 5): (a) CCl4 group; (b) PB2(M) group, treated with PB2 100 mg/kg by gavage during the 5th‐8th weeks; (c) cyclopamine group, treated with cyclopamine 30 mg/kg by gavage during the 5th‐8th weeks^6^; (d) PB2 + cyclopamine group, treated with PB2 100 mg/kg and cyclopamine 30 mg/kg during the 5th‐8th weeks.

Twenty‐four hours after the last treatment, all of the mice were anaesthetized with pentobarbital intraperitoneally. Blood samples were collected and kept at −80°C. Some of the liver tissues were cut and stored at −80°C; other tissue samples were immersed in 4% paraformaldehyde.

### 2.3 Cell culture and CCK8 assay

The human immortal LX2 cell line was cultured in high glucose DMEM with 10% FBS, 100 U/mL of penicillin and 100 g/mL of streptomycin. The apparent logarithmic phase cells were seeded in 96‐well plates for 48 hours, then PB2 was added at concentrations of 10, 20, 40, 80, 160 or 320 μM for 24 hours, and the cytotoxicity analysis was performed. Cell viability was then measured with the CCK8 assay. The in vitro experiments were conducted using the following four groups: (a) NC group, LX2 cells without any treatment; (b‐d) L, M and H groups, respectively, LX2 cells were treated with 60, 80 and 100 μM PB2, respectively; (e) cyclopamine group, LX2 cells were treated with 10 μM cyclopamine^6^; and (f) PB2 (M) + SAG group, LX2 cells were treated with 100 nM SAG.^27^, ^28^ All of the experiments were performed in triplicate.

### 2.4 Flow cytometry analysis

Cells were seeded in 6‐well plates for 48 hours and then treated with different concentrations of PB2 for 24 hours. The Annexin V‐FITC/PI apoptosis detection kit and a flow cytometer (Beckman Coulter, Inc) were used for detection of cell apoptosis. Annexin V‐FITC positive (with or without PI‐positive) cells were regarded as apoptotic (early‐phase or late phase) cells using the FlowJo software (version 10; FlowJo LLC).

### 2.5 Biochemical assays

Detection of serum ALT, AST and liver hydroxyproline concentrations were determined according to the manufacturer's protocols.

### 2.6 Histopathology

Paraformaldehyde‐immersed liver tissues were embedded in paraffin and cut into 3‐μm thick sections. For haematoxylin and eosin (H & E) staining, the slices were stained with H for 10 minutes and E for 5 minutes to demonstrate the liver injuries. Masson's trichrome and Sirius Red staining were used according to previous studies.^25^, ^29^ Five random fields of view for each slice were captured and analysed in the experiment.

### 2.7 Reverse transcription‐polymerase chain reaction and quantitative real‐time PCR

The total RNA from LX2 cells was extracted by TRIzol, and reverse transcription‐polymerase chain reaction (RT‐PCR) was performed. The primers used for quantitative real‐time PCR (qRT‐PCR) are listed in Table 2 and were determined using a 7900HT Fast PCR System (Applied Biosystems). The results were quantified using the 2^−ΔΔCt^ method.^30^

**Table 2:** Primers used in the study

Genes name	Forward (5′‐3′)	Reverse (5′‐3′)
α‐SMA	AAAAGACAGCTACGTGGGTGA	GCCATGTTCTATCGGGTACTTC
Col‐1α1	GAGGGCCAAGACGAAGACATC	CAGATCACGTCATCGCACAAC
Col‐1α2	GTTGCTGCTTGCAGTAACCTT	AGGGCCAAGTCCAACTCCTT
TGF‐β1	GGCCAGATCCTGTCCAAGC	GTGGGTTTCCACCATTAGCAC
VEGF‐A	AGGGCAGAATCATCACGAAGT	AGGGTCTCGATTGGATGGCA
SMO	GAAGTGCCCTTGGTTCGGA	GCAGGGTAGCGATTCGAGTT
GLI1	AGCGTGAGCCTGAATCTGTG	CAGCATGTACTGGGCTTTGAA
HIF‐1α	GAACGTCGAAAAGAAAAGTCTCG	CCTTATCAAGATGCGAACTCACA
β‐actin	CATGTACGTTGCTATCCAGGC	CTCCTTAATGTCACGCACGAT

### 2.8 Western blot analysis

Total protein was extracted using RIPA lysis buffer and quantified with a BCA kit. Eighty micrograms of protein were loaded per well on 7.5%‐12.5% SDS‐PAGE for electrophoresis, then transferred to PVDF membranes and blocked with 5% non‐fat milk, following by incubation with primary antibodies overnight. The next day, the membranes were incubated with secondary antibodies and detected using Odyssey (Licor). Quantitative analysis of Western blots was conducted by ImageJ software.

### 2.9 Immunohistochemistry staining

After dewaxed and rehydrated, the sections were performed with an antigen retrieval technique. About 3% hydrogen peroxide and 5% BSA were used to block the endogenous and non‐specific binding sites. Then, the slices were incubated with primary antibodies at 4°C. The next day, slides were incubated with secondary antibody and then counterstained with H&E and observed. The brown‐stained cells were regarded as positive cells, and the positive rates were calculated using Image‐Pro Plus software 6.0.

### 2.10 Immunofluorescence staining

The treated LX2 cells slices were fixed with 4% paraformaldehyde and incubated with primary antibodies against α‐SMA, VEGF‐A or GLI1 at 4℃. The next day, the slices were incubated with fluorescence secondary antibody and DAPI, and then mounted with anti‐fluorescence quenching sealant and observed with an inverted fluorescence microscope (Leica DMIRB).

### 2.11 Tubulogenesis assay

The tubulogenesis assay was designed with the guidance of a previous study.^6^ Briefly, the NC, PB2 (M), Sora and PB2 + Sora groups of LX2 cells were pretreated with related drugs for 24 hours and starved for 8 hours before use. Fifty microlitres of growth factor‐reduced Matrigel were added to 96‐well plates and placed in a 37℃ incubator for 30 minutes. Then, the pretreated LX2 cells were resuspended with FBS‐free DMEM and added to the Matrigel at a density of 2 × 10^4^/well. The results were observed and captured by microscopy at 2, 3, 4, 5, 6, 8, 10 and 24 hours after seeding.

### 2.12 Statistical analysis

All data are reported as mean ± standard deviation (SD) and imaged using GraphPad Prism 6 software (GraphPad Software). The results were analysed with Student's *t* test and one‐way analysis of variance, followed by Tukey's post hoc test. Statistically significance was defined as *P* < .05.

## 3 RESULTS

### 3.1 PB2 has no toxicity on liver function and liver pathology

**Figure 1:**
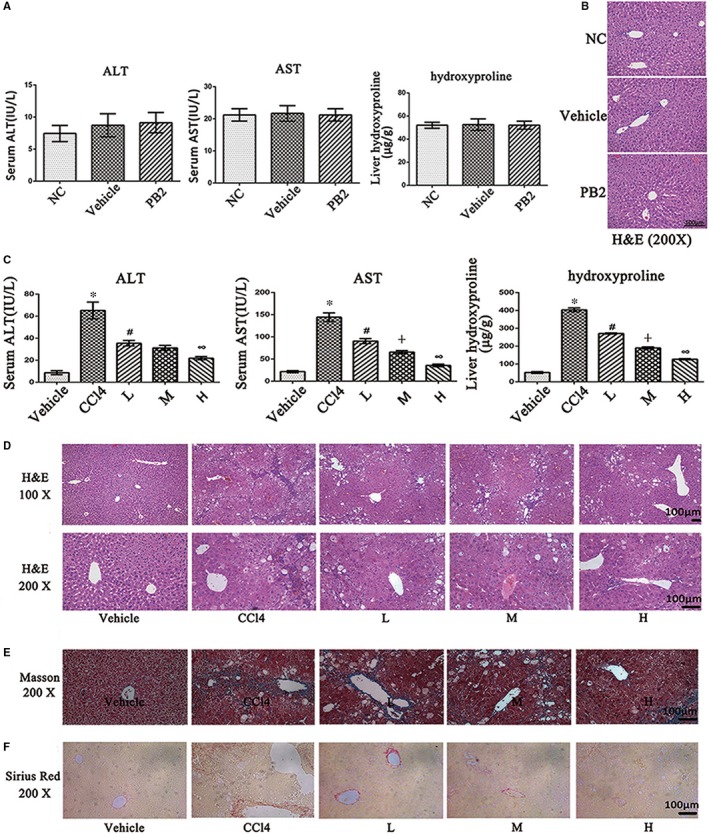
Effects of PB2 on CCl4‐induced liver fibrosis in mice. A, The serum ALT and AST levels and liver hydroxyproline concentrations. Data are presented as the mean ± SD (n = 6, from three independent experiments). B, The H & E staining of liver tissues in the NC, vehicle and PB2 groups (original magnification, 200×). The structures were clear, and the hepatocytes were well‐preserved with clear cytoplasm and prominent nuclei and nucleoli. C, The serum ALT and AST levels and liver hydroxyproline concentrations. Data are presented as the mean ± SD (n = 6, from three independent experiments). * Indicates *P* < .05 vs the vehicle group; # indicates *P* < .05 vs the CCl4 group; ┼ indicates *P* < .05 vs the L group; ∞ indicates *P* < .05 vs the M group (D) The H & E staining of liver tissues (original magnification, 100× and 200×). There was a rearrangement of liver lobular structures, a formation of peri‐cellular collagen deposition, and diffuse swelling of hepatocytes and infiltration of inflammatory cells in the CCl4 group. PB2 can effectively relieve the pathologic injuries. E, Masson's trichrome staining of liver tissues. The blue‐stained areas referred to ECM deposition (original magnification, 200×). F, Sirius Red staining of liver tissues. The red‐stained areas indicated type Ⅰ collagen deposition (original magnification, ×200)

To study the toxicity of PB2 and vehicle oil on liver, we conducted a preliminary study to examine the safety of PB2 on liver function in mice. As shown in Figure 1A, the serum ALT and AST levels, which are usually regarded as biomarkers of liver injury, were not elevated in the vehicle and PB2 (150 mg/kg) groups when compared to the NC group. The liver hydroxyproline concentration, which is used as an indirect measurement of tissue collagen content, did not differ between these three groups as well. H&E staining of liver slices in the three groups revealed normal morphology (Figure 1B). These results indicated that vehicle oil and PB2 (at the highest test concentration) had no toxic effects on liver function and liver pathology. Therefore, the vehicle group served as the control group in subsequent animal experiments.

### 3.2 PB2 relieves liver injury and fibrogenesis in CCl4‐induced liver fibrosis in mice

Because PB2 is safe, we then established a CCl4‐induced liver fibrosis murine model to determine the effect of PB2 in liver fibrosis. The results in Figure 1C show that the serum levels of ALT and AST were dramatically elevated in the CCl4 group when compared to the vehicle group with respect to the occurrence of liver injuries. The liver hydroxyproline concentration was also increased in the CCl4 group; however, treatment of PB2 effectively reduced the levels of liver enzymes and hydroxyproline in a dose‐dependent manner. The H&E staining showed that in the vehicle group, the liver tissues were well‐constructed and the hepatocytes were well‐preserved with clear cytoplasm and a prominent nucleus and nucleolus. In the CCl4 group, normal liver structure was replaced by a rearrangement of liver lobular structures and a formation of peri‐cellular collagen deposition. There was diffuse swelling of hepatocytes and infiltration of inflammatory cells. PB2 treatment also reduced the pathological changes of liver tissues (Figure 1D). Masson's trichrome and Sirius Red staining, which are used to detect collagen deposition, also revealed the same results as H&E staining (Figure 1E,F). These results confirmed the successful establishment of a liver fibrosis model and indicated that PB2 treatment can attenuate liver fibrogenesis in mice in a dose‐dependent manner.

### 3.3 PB2 inhibits the angiogenesis during liver fibrosis

**Figure 2:**
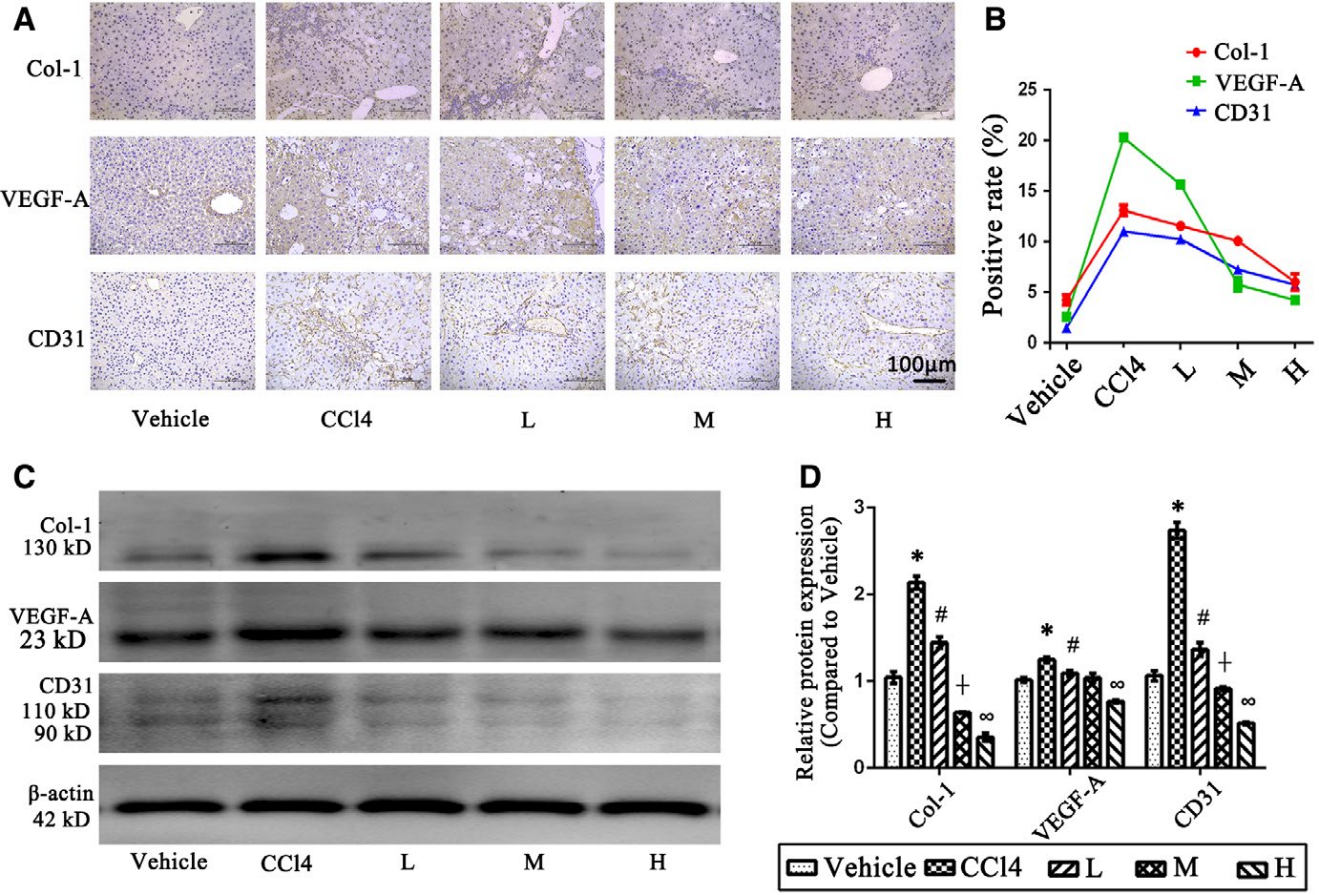
Effect of PB2 on the ECM deposition and angiogenesis in CCl4‐induced liver fibrosis tissues. A, IHC staining of Col‐1, VEGF‐A and CD31 in liver tissues (original magnification, 200×). The brown‐stained areas were considered to be positive. B, The quantitative analysis of IHC results. C & D, The expression of Col‐1, VEGF‐A and CD31 protein in liver tissues and the quantitative analysis of Western blot results. Data are presented as the mean ± SD (n = 6, from three independent experiments). * indicates *P* < .05 vs the vehicle group; # indicates *P* < .05 vs the CCl4 group, ┼ indicates *P* < .05 vs the L group and ∞ indicates *P* < .05 vs the M group

As reported, angiogenesis is a remarkable pathological manifestation of liver fibrosis and always develops in parallel with fibrogenesis in chronic liver disease.^6^, ^7^ We used immunohistochemistry (IHC) staining to detect the expression of Col‐1 (a vital marker for ECM deposition), VEGF‐A (an effective cytokine that promotes angiogenesis) and CD31 (a marker for the formation of new vessels) in liver tissues. As shown in Figure 2A,B, the expression of Col‐1, VEGF‐A and CD31 was limited in the vehicle group, and the positive rate of these three markers was higher in the CCl4 group. Thus, there was significant ECM deposition and angiogenesis in the liver fibrosis model, and the statistical analysis of IHC staining in Figure 2B confirmed that angiogenesis was positively correlated with fibrogenesis. In the PB2 treatment groups, the IHC positive rates of Col‐1, VEGF‐A and CD31 were significantly reduced in a dose‐dependent fashion. Western blot analysis of liver tissues also demonstrated the same results as IHC staining (Figure 2C,D). Overall, these results showed that PB2 ameliorated fibrogenesis and angiogenesis in liver tissues.

### 3.4 PB2 suppresses proliferation and activation, and promotes apoptosis of LX2 cells

**Figure 3:**
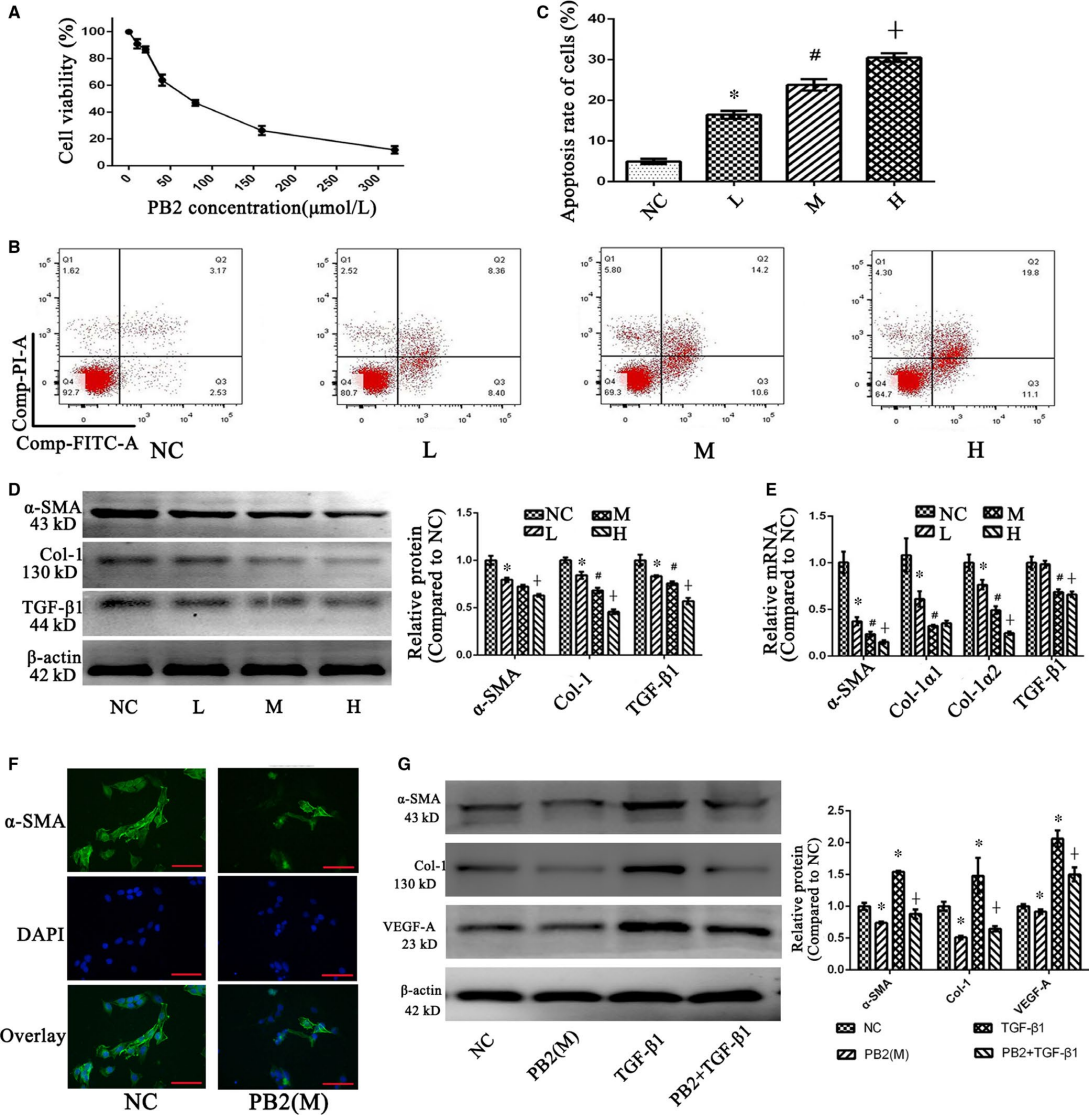
Effects of PB2 on the proliferation, apoptosis and activation of LX2 cells. A, The CCK8 assay was used to determine the effects of PB2 on the viability of LX2 cells. B & C, Flow cytometry detection of apoptosis and apoptosis rate of LX2 cells. D, The expression of α‐SMA, Col‐1 and TGF‐β1 protein in LX2 cells. E, The levels of α‐SMA, Col‐1α1, Col‐1α2 and TGF‐β1 mRNA in LX2 cells. All of the experiments were repeated in triplicate. Data are presented as the mean ± SD from three independent experiments. * Indicates *P* < .05 vs the NC group; # indicates *P* < .05 vs the L group; and ┼ indicates *P* < .05 vs the M group. F, IF staining of α‐SMA in LX2 cells in the NC and PB2 (M) groups (original magnification, ×400; bar: 50 μm). G, Effects of TGF‐β1 and PB2 co‐treatment on LX2 cells' activation and angiogenesis. PB2 was effective to reverse the activation effect of TGF‐β1 on LX2 cells. * Indicates *P* < .05 vs the NC group; ┼ indicates *P* < .05 vs the TGF‐β1 group

To further investigate the protective mechanism of PB2 in liver fibrosis, a human immortal HSC cell line (LX2 cells) was used in this study. The CCK8 assay was used to measure the toxicity of PB2 in LX2 cells (Figure 3A). PB2 inhibited the viability of LX2 cells in a dose‐dependent manner, and the half‐maximal inhibitory concentration (IC50) was 82.56 μM. In a subsequent in vitro experiment, 60, 80 and 100 μM of PB2 were used as a different concentration gradient to treat LX2 cells and defined as the L, M and H groups, respectively. Flow cytometry analysis of apoptosis in Figure 3B,C showed that PB2 promoted apoptosis the LX2 cells. Via the inhibition of proliferation and promotion of apoptosis, the number of LX2 cells was decreased. The activation of HSCs by TGF‐β1 is an important process during fibrogenesis, leading to the excessive production of ECM. The qRT‐PCR and Western blot results showed that the expression of α‐SMA (a marker of activated HSCs), Col‐1, and TGF‐β1 mRNA and protein was reduced in the L, M and H groups, indicating that PB2 treatment inhibited the activation and ECM production in HSCs (Figure 3D,E). The immunofluorescence (IF) stain of α‐SMA in LX2 cells also confirmed inhibition of α‐SMA expression in HSCs, which was consistent with the qRT‐PCR and Western blot results (Figure 3F). Moreover, to furtherly verify the role of PB2 during the inhibition of HSCs activation, exogenous TGF‐β1 (10 ng/mL) was used to activated LX2 cells.^31^ As the results shown in Figure 3G, in TGF‐β1 treated group, the expression of α‐SMA was enhanced in LX2 cells when compared to NC group, and PB2 treatment can inhibit the expression of α‐SMA. However, if LX2 cells were administrated with both TGF‐β1 and PB2, then the overexpression of α‐SMA induced by TGF‐β1 can also be depressed, which indicated PB2 was effective to reverse the activation effect of TGF‐β1. The expression of Col‐1 and VEGF‐A was similar to α‐SMA after both TGF‐β1 and PB2 treatment. These results declared that PB2 can suppress the proliferation and activation, and promote the apoptosis of LX2 cells.

### 3.5 PB2 inhibits the angiogenesis ability of LX2 cells

**Figure 4:**
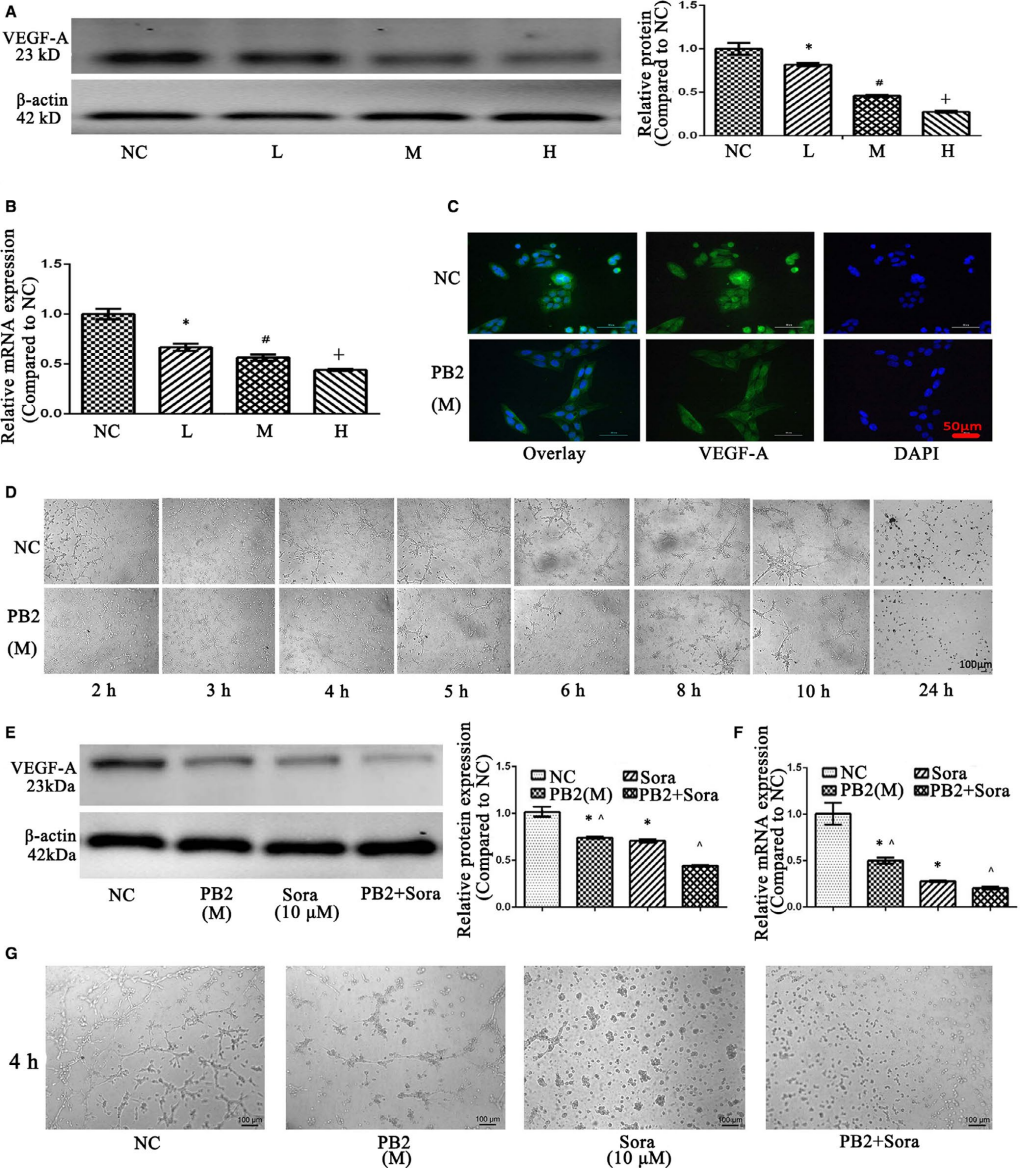
Effects of PB2 on the angiogenesis ability of LX2 cells. A, The expression of VEGF‐A protein in LX2 cells. B, The levels of VEGF‐A mRNA in LX2 cells. C, IF staining of VEGF‐A in LX2 cells (original magnification, 400×). D, A tubulogenesis assay was used to detect the angiogenesis ability of LX2 cells (original magnification, 100×). PB2 inhibited the formation and size of closed rings or pro‐angiogenic structures in LX2 cells. E, Effect of sorafenib on the protein expression of VEGF‐A. F, Effect of sorafenib on the transcription of VEGF‐A. G, Effect of sorafenib on the angiogenesis ability of LX2. All of the experiments were repeated in triplicate. Data are presented as the mean ± SD from three independent experiments. * Indicates *P* < .05 vs the NC group; # indicates *P* < .05 vs the L group; ┼ indicates *P* < .05 vs the M group; and ^ indicates *P* < .05 vs the Sora group

Hepatic stellate cells have been shown to regulate angiogenesis during liver fibrosis by secreting strong stimuli of angiogenesis, such as VEGF‐A, angiopoietin 1, placental growth factor and epidermal growth factor chemokine receptor l.^32^, ^33^ VEGF‐A is the most potent stimulus and promotes the proliferation, migration, differentiation and tubulogenesis of vascular endothelial cells, thus giving rise to angiogenesis in liver fibrosis. Therefore, we used different concentrations of PB2 to treat LX2 cells and analysed the angiogenesis ability of HSCs. The Western blot and qRT‐PCR results showed that the translation and transcription of VEGF‐A were inhibited by PB2 treatment in a dose‐dependent manner (Figure 4A,B). IF staining of VEGF‐A in the NC and M groups also verified that the expression of VEGF‐A was suppressed by PB2 in LX2 cells (Figure 4C). Moreover, the tubulogenesis assay, which can examine the angiogenesis ability of LX2 cells, revealed that the tubulogenesis ability of LX2 cells fluctuated with time and peaked at 5 hours in the NC group (Figure 4D). In the M group, however, PB2 treatment inhibited the tubulogenesis ability of LX2 cells, which was reflected by fewer and smaller, but earlier elimination of cavity formation.

To further verify the anti‐angiogenesis ability of PB2, we used sorafenib, which can inhibit the neovascularization in liver cancers, to compare the angiogenesis inhibition effect between PB2 and sorafenib. The dosage of sorafenib was 10 μM, which was determined according to previous studies.^34^, ^35^, ^36^ As the results shown in Figure 4E, the protein expression of VEGF‐A was decreased in both PB2(M) and Sora group, and was much lower in the PB2 + Sora group. In Figure 4F, the qRT‐PCR results are shown to be consistent with the Western blot results. In addition, the tubulogenesis assay in Figure 4G demonstrated that both PB2 and sorafenib inhibited the tubulogenesis ability of LX2 cells at 4 hours. These indicated that PB2 was as effective in inhibiting the angiogenesis process in LX2 cells as sorafenib, and the combined treatment of PB2 and sorafenib enhanced the angiogenesis inhibitory effect of sorafenib.

In conclusion, these results confirmed the angiogenesis ability of LX2 cells and indicated that PB2 effectively inhibited the angiogenesis ability of LX2 cells by suppressing the expression of VEGF‐A and tubulogenesis.

### 3.6 PB2 reverses liver fibrosis by down‐regulating the Hh pathway in vivo and in vitro

**Figure 5:**
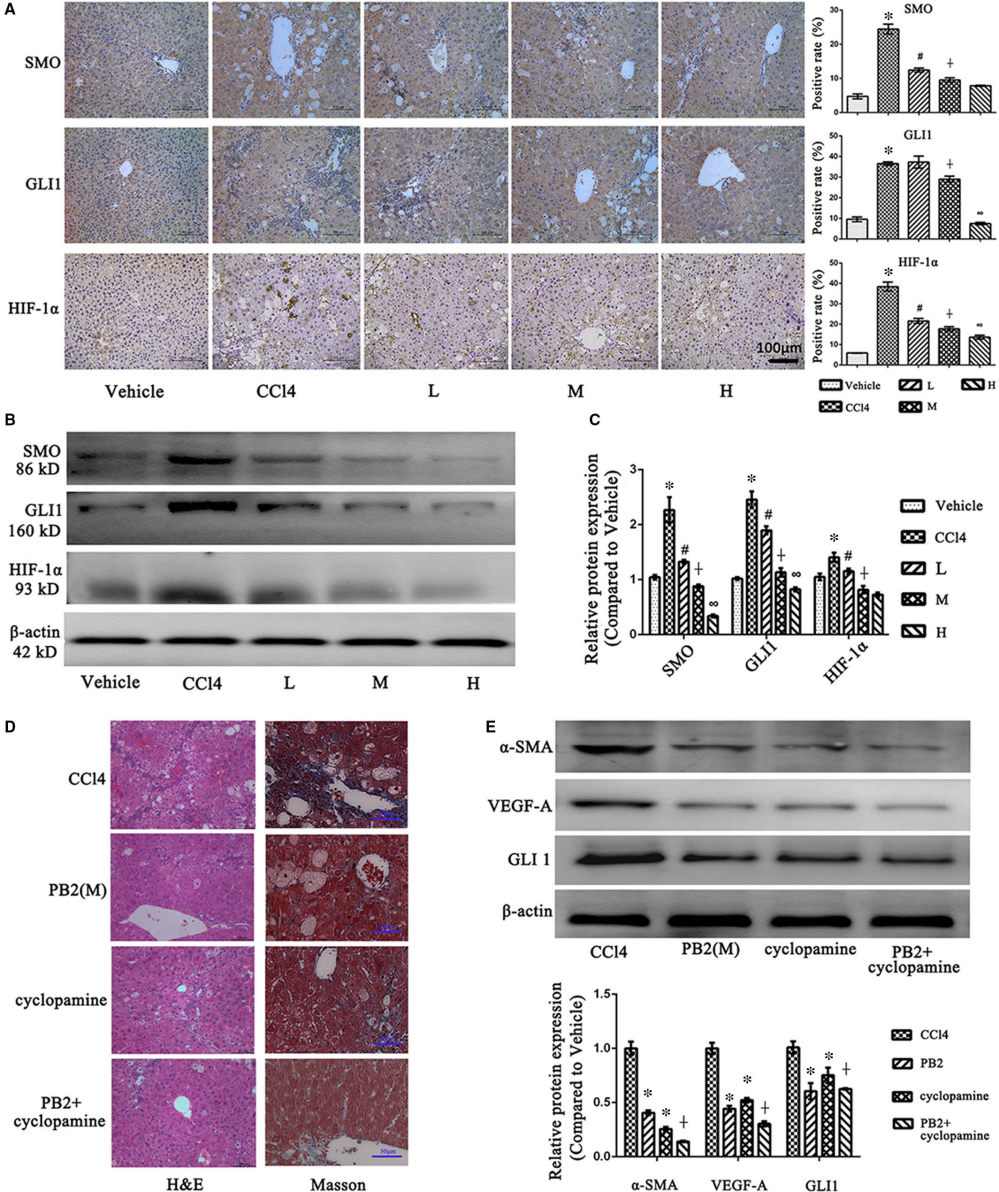
Effects of PB2 on the Hh pathway in CCl4‐induced liver fibrosis in mice. A, IHC staining and quantitative analysis of SMO, GLI1 and HIF‐1α in liver tissues (original magnification, ×200). B, The expression of SMO, GLI1 and HIF‐1α protein in liver tissues. C, The quantitative analysis of Western blot results. Data are presented as the mean ± SD (n = 6, from three independent experiments). * Indicates *P* < .05 vs the vehicle group; # indicates *P* < .05 vs the CCl4 group; ┼ indicates *P* < .05 vs the L group; and ∞indicates *P* < .05 vs the M group. D, The H&E staining and Masson's trichrome staining of liver tissues after cyclopamine treatment in vivo. E, The Western blotting analysis of α‐SMA, VEGF‐A and GLI1 after cyclopamine treatment in vivo. Data are presented as the mean ± SD (n = 5). * Indicates *P* < .05 vs the CCl4 group; ┼ indicates *P* < .05 vs the cyclopamine group

To further determine the molecular mechanism of PB2 in liver fibrosis, the Hh pathway aroused our attention. SMO and GLI1 are two important molecules in the Hh pathway. In the CCl4‐induced liver fibrosis model in mice, IHC staining showed that in the vehicle group there was minimal expression of SMO and GLI1 in normal liver tissues, but increased expression in the CCl4 group (Figure 5A). In the PB2‐treated groups, expression of SMO and GLI1 was decreased. The Western blot results were consistent with the IHC staining results (Figures 5B,C). HIF‐1α is not only a target gene of the Hh pathway, but a neovascularization‐driven factor, playing a role in angiogenesis and liver injury during liver fibrosis.^6^, ^32^ The IHC staining and Western blot results revealed that there was high expression of HIF‐1α in fibrotic liver tissues, and the expression could be inhibited by PB2 (Figure 5A‐C).

Moreover, we used the Hh pathway inhibitor cyclopamine to compare the role of PB2 and cyclopamine in vivo. The results in Figure 5C demonstrated that both PB2 and cyclopamine can relieve the pathological injuries and collagen deposition in liver tissues, and if treated with both PB2 and cyclopamine, the improvement was more effective than mono‐treatment. The Western blotting analysis of α‐SMA, VEGF‐A and GLI1 in liver tissues also revealed that PB2 and cyclopamine co‐treatment was better to relief HSCs activation, angiogenesis and the Hh pathway than the mono‐treatment. These results indicated that PB2 was effective to reduced liver fibrosis as Hh inhibitor cyclopamine did, and PB2 and cyclopamine co‐treatment exhibited a better effect than mono‐treatment.

**Figure 6:**
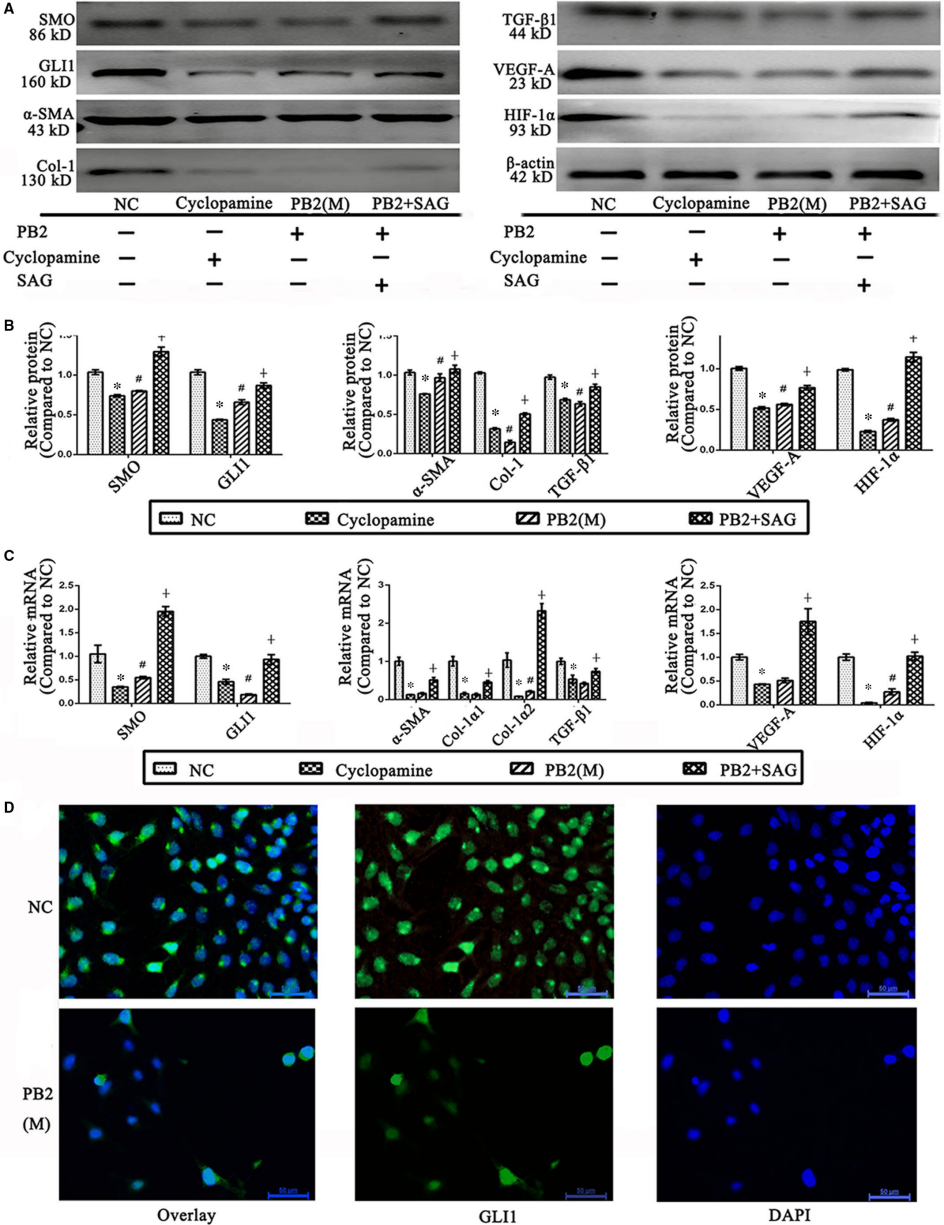
Effects of PB2 on the Hh pathway in LX2 cells. A, Effects of cyclopamine, PB2, and SAG on the Hh pathway, and the activation and angiogenesis ability of LX2 cells. B, The quantitative analysis of Western blot results. C, The levels of SMO, GLI1, α‐SMA, Col‐1α1, Col‐1α2, TGF‐β1, VEGF‐A and HIF‐1α mRNA in LX2 cells. D, IF staining of GLI1 in LX2 cells (original magnification, 400×). All of the experiments were repeated in triplicate. Data are presented as the mean ± SD from three independent experiments. * Indicates *P* < .05 vs the NC group; # indicates *P* < .05 vs the L group; and ┼ indicates *P* < .05 vs the M group

Immunofluorescence staining of GLI1 was positive in the cytoplasm and nuclei of LX2 cells, which indicated that the Hh pathway was activated in HSCs (Figure 6D); however, if the LX2 cells were treated with PB2, the expression and translocation of GLI1 from the cytoplasm to the nucleus was inhibited. To verify whether or not the Hh pathway was the target of PB2 action, we used the cyclopamine (a SMO inhibitor) and SAG (a SMO agonist). As shown in Figure 6A‐C, the Western blot and qRT‐PCR results revealed that the expression of SMO and GLI1 was high in the NC group. In the cyclopamine group, if the LX2 cells were treated with a Hh pathway inhibitor, the expression of SMO and Hh was dramatically reduced. In the PB2 (M) group, the inhibitory effects of PB2 on SMO and GLI1 were almost the same as cyclopamine, while the inhibition was significantly reversed by SAG. These findings demonstrated that the Hh pathway was the target action site of PB2 in liver fibrosis. The detection of α‐SMA, Col‐1, TGF‐β1, VEGF‐A and HIF‐1α by Western blot and qRT‐PCR also showed that by inhibiting the Hh pathway by cyclopamine, the activation and angiogenesis ability of LX2 cells were significantly inhibited. Overall, these analyses confirmed that PB2 inhibited the Hh pathway to inhibit the activation, ECM production and angiogenesis ability of HSCs during liver fibrosis in vivo and in vitro.

## 4 DISCUSSION

Liver fibrosis is a serious health problem worldwide because liver fibrosis can ultimately progress into end‐stage liver cirrhosis and even HCC.^3^ The reversibility of liver fibrosis is commonly recognized if the fibrogenesis stimulus is removed.^2^, ^37^ In many chronic liver diseases, however, the elimination of primary fibrogenesis factors cannot be eliminated. Various therapeutic measures have been developed to reverse liver fibrosis, such as immunosuppressants, anti‐inflammatory agents, PPAR‐γ agonists, pan‐caspase inhibitors and Hh inhibitors.^37^, ^38^, ^39^, ^40^

PB2 (epicatechin‐(4h‐8)‐epicatechin), which is rich in grape seeds, apples and cacao beans, is well‐known for its anti‐inflammatory and anti‐tumour properties.^41^, ^42^ Previously, PB2 has been reported to protect against CCl4‐induced acute liver injury by suppressing the inflammatory response and apoptosis in liver tissues.^42^ There are no studies about the effects of PB2 on chronic liver injuries to date. Therefore, we used CCl4 to induce a liver fibrosis murine model and examine the effects of PB2 on chronic liver injuries. The results in Figure 1 revealed that PB2 was safe for the long‐term use in mice, and 50, 100 and 150 mg/kg could inhibit and even reverse the progression of liver fibrosis.

Liver fibrosis is characterized by the over‐deposition of ECM, and the activation of HSCs by TGF‐β1, PDGF and EGF is the key link to the pathogenesis of liver fibrosis.^5^, ^43^ In the normal liver, HSCs maintain the production and degradation of ECM to maintain the balance of ECM deposition; however, in liver fibrosis the excessive activation of qHSCs to aHSCs contributes to the over‐deposition of ECM, especially collagen.^3^, ^4^ The results in Figure 2 showed that the expression of Col‐1 is significantly inhibited by PB2, which further confirmed the protective effect of PB2 on liver fibrosis. Subsequently, we used a human immortal HSC cell line (LX2) to more thoroughly investigate the protective mechanism. We found that the proliferation and activation of LX2 cells were restrained by PB2, and ECM production of LX2 cells was also decreased. In addition, flow cytometry demonstrated that PB2 promotes apoptosis of LX2 cells. In summary, we showed that PB2 decreased the number of LX2 cells via inhibition of proliferation and induction of apoptosis, and inhibited the activation and ECM production ability of relict LX2 cells to exert an anti‐fibrotic effect.

**Figure 7:**
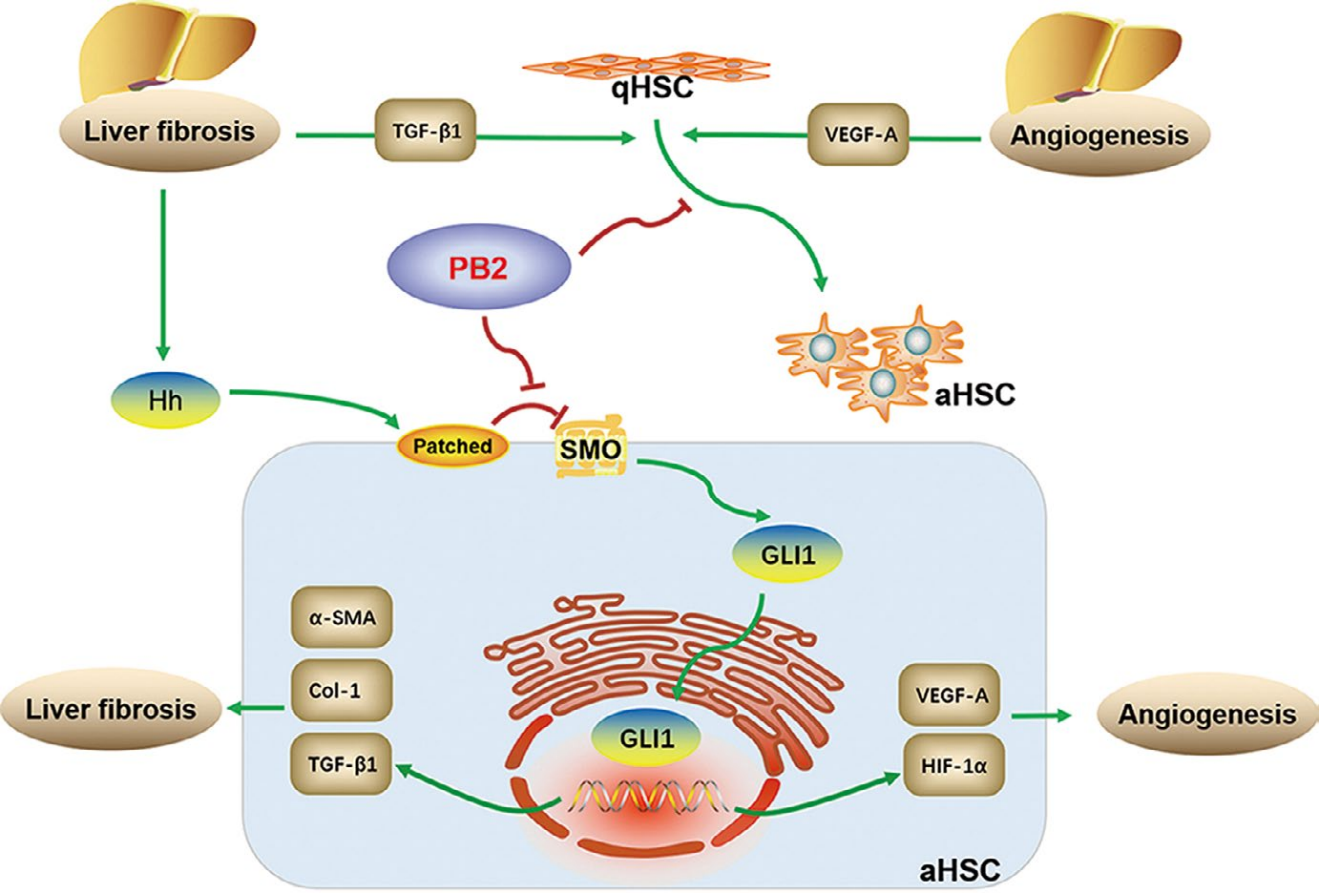
Mechanism of PB2 on the protective effect in liver fibrosis. Liver fibrosis is mainly initiated by the activation of HSCs. The injured liver tissues can release Hh ligands to activate the Hh pathway, leading to the activation of HSCs, ECM deposition and angiogenesis during liver fibrosis. In the current study, PB2 directly inhibited the Hh signalling pathway, therefore down‐regulating the transcription of VEGF‐A, HIF‐1α, α‐SMA, Col‐1 and TGF‐β1 in HSCs to suppress the activation, ECM production and angiogenesis of HSCs, leading to the reversal of liver fibrosis

We also showed that PB2 ameliorated angiogenesis of HSCs in vivo and in vitro, which was reflected by the inhibition of VEGF‐A expression and tubulogenesis ability of LX2 cells. Many studies have shown that angiogenesis is associated with fibrogenesis, and angiogenesis is thus a promising therapeutic target of liver fibrosis.^44^, ^45^ There are close connections between the activation of HSCs and angiogenesis. First, aHSCs possess angiogenesis ability by secreting VEGF‐A and angiopoietin 1, which can directly stimulate the sinus endothelial cells and provoke the formation and stability of neovascularity.^33^, ^46^, ^47^ VEGF‐A can also provide positive feedback to promote the proliferation and activation of HSCs.^48^ Second, angiogenesis is a hypoxia‐stimulated process in liver fibrosis because the over‐deposition of ECM disrupts the architecture and gives rise to tissue hypoxia.^6^, ^32^ The aHSCs enhance the expression of HIF‐1α, leading to the transcription of an increase in angiogenesis genes.^49^, ^50^ Third, the HSCs can also express lots of CXC chemokines, which can manipulate angiogenesis during initiation and progression of fibrogenesis.^51^ Fourth, HSCs can also regulate the blood flow of vessels to modulate hepatic microvascular dynamics.^52^ Fifth, angiogenesis during liver fibrosis is invalid blood vessels, which are deficient in improving but will accelerate tissue hypoxia. In contrast, the angiogenesis will exacerbate structure turbulence and hypoxia, which can promote activation and ECM production of HSCs.^33^, ^53^ In the current study, PB2 inhibited the angiogenesis in LX2 cells and liver tissues, which also accounted for the anti‐fibrotic effect of PB2 (Figure 7).

The precise action target of PB2 was further investigated. As reported, the Hh pathway plays an important role during liver fibrosis, including the activation, ECM secretion and metabolism of HSCs to control liver fibrosis.^54^, ^55^, ^56^ The Hh signalling pathway is initiated by the secretion of ligands (including sonic hedgehog [Shh], Indian hedgehog [Ihh] and desert hedgehog [Dhh]).^57^ The HSCs are Hh‐responsive cells and can receive the Hh ligands auto‐ or pan‐secreted by injured hepatocytes, HSCs, liver progenitors and some types of resident lymphocytes, which can then interact with the receptor, Patched.^19^, ^42^ Patched will eliminate the inhibitory effect on SMO, which will then promote the activation and nuclear translocation of the transcription factor, GLI1, resulting in the expression of Hh‐target genes, such as VEGF‐1, HIF‐1α, Gli1, SMO and Bcl‐2.^58^, ^59^ The Hh pathway also plays a role in angiogenesis in liver fibrosis, as well as hepatocarcinoma.^60^ Therefore, in the current study we used the SMO inhibitor, cyclopamine, and the SMO agonist, SAG, to determine the role of PB2 on the Hh pathway in liver fibrosis. Indeed, PB2 possessed almost the same inhibitory effect on the Hh pathway as cyclopamine, and the inhibitory effect was reversed by SAG. These findings primarily indicated that the Hh pathway is the action target of PB2 on liver fibrosis. In addition, inhibition of the Hh pathway by cyclopamine and PB2 can inhibit the activation, ECM production and angiogenesis effect of HSCs, probably via the down‐regulation of VEGF‐A, HIF‐1α, α‐SMA, Col‐1 and TGF‐β1 transcription in LX2 cells.

In addition, because liver fibrosis may progress into liver cirrhosis and even hepatocarcinoma, and angiogenesis is a crucial characteristic of cancer, the anti‐angiogenesis property of PB2 suggested a possible therapeutic role in hepatocarcinoma as well.

In conclusion, we confirmed that PB2 inhibited the Hh signalling pathway and therefore down‐regulated the transcription of VEGF‐A, HIF‐1α, α‐SMA, Col‐1 and TGF‐β1 in HSCs to suppress activation, ECM production and angiogenesis, thus reversing the progression of liver fibrosis.

## CONFLICT OF INTEREST

The authors confirm that there are no conflicts of interest.

## AUTHOR CONTRIBUTIONS

J. Feng and J. Li contributed to the design of research; J. Feng, L. Wu, J. Ji and K. Chen performed the data analysis; J. Feng, Q. Yu, S. Li and T. Liu helped perform the cellular experiments; J. Zhang, J. Chen and Y. Zhou helped perform the animal experiments; Y. Mao, F. Wang and W. Dai conducted some other experiments; J. Feng wrote the manuscript; X. Fan, J. Wu and Chuanyong Guo edited the manuscript.

## Data Availability

The datasets generated during and/or analysed during the current study are available from the corresponding author on reasonable request.
